# Upper Urinary Tract Stereotactic Body Radiotherapy Using a 1.5 Tesla Magnetic Resonance Imaging-Guided Linear Accelerator: Workflow and Physics Considerations

**DOI:** 10.3390/cancers16233987

**Published:** 2024-11-27

**Authors:** Yao Zhao, Adrian Cozma, Yao Ding, Luis Augusto Perles, Reza Reiazi, Xinru Chen, Anthony Kang, Surendra Prajapati, Henry Yu, Ergys David Subashi, Kristy Brock, Jihong Wang, Sam Beddar, Belinda Lee, Mustefa Mohammedsaid, Sian Cooper, Rosalyne Westley, Alison Tree, Osama Mohamad, Comron Hassanzadeh, Henry Mok, Seungtaek Choi, Chad Tang, Jinzhong Yang

**Affiliations:** 1 Department of Radiation Physics, The University of Texas MD Anderson Cancer Center, Houston, TX 77030, USA; yzhao15@mdandrson.org (Y.Z.); yding1@mdanderson.org (Y.D.); laperles@mdanderson.org (L.A.P.); rreiazi@mdanderson.org (R.R.); xchen20@mdanderson.org (X.C.); anthony.m.kang@uth.tmc.edu (A.K.); sprajapati1@mdanderson.org (S.P.); zyu1@mdanderson.org (H.Y.); edsubashi@mdanderson.org (E.D.S.); kkbrock@mdanderson.org (K.B.); jihong.wang@mdanderson.org (J.W.); abeddar@mdanderson.org (S.B.); blee5@mdanderson.org (B.L.); 2 Department of Radiation Oncology, The University of Texas MD Anderson Cancer Center, Houston, TX 77030, USA; adrian.i.cozma@gmail.com (A.C.); mmsaid@mdanderson.org (M.M.); omohamad@mdanderson.org (O.M.); cjhassanzadeh@mdanderson.org (C.H.); hmok@mdanderson.org (H.M.); stchoi@mdanderson.org (S.C.); 3 The University of Texas MD Anderson Cancer Center UTHealth Houston Graduate School of Biomedical Sciences, Houston, TX 77030, USA; 4 Department of Imaging Physics, The University of Texas MD Anderson Cancer Center, Houston, TX 77030, USA; 5 The Royal Marsden Hospital, Institute of Cancer Research, London SW3 6JJ, UK; sian.cooper@rmh.nhs.uk (S.C.); rosalyne.westley@rmh.nhs.uk (R.W.); alison.tree@icr.ac.uk (A.T.)

**Keywords:** MR-Linac, MRgRT, kidney, SBRT, RCC, UTUC

## Abstract

**Background/Objectives:** Advancements in radiotherapy technology now enable the delivery of ablative doses to targets in the upper urinary tract, including primary renal cell carcinoma (RCC) or upper tract urothelial carcinomas (UTUC), and secondary involvement by other histologies. Magnetic resonance imaging-guided linear accelerators (MR-Linacs) have shown promise to further improve the precision and adaptability of stereotactic body radiotherapy (SBRT). **Methods:** This single-institution retrospective study analyzed 34 patients (31 with upper urinary tract non-metastatic primaries [RCC or UTUC] and 3 with metastases of non-genitourinary histology) who received SBRT from August 2020 through September 2024 using a 1.5 Tesla MR-Linac system. Treatment plans were adjusted by using [online settings] for “adapt-to-position” (ATP) and “adapt-to-shape” (ATS) strategies for anatomic changes that developed during treatment; compression belts were used for motion management. **Results:** The median duration of treatment was 56 min overall and was significantly shorter using the adapt-to-position (ATP) (median 54 min, range 38–97 min) in comparison with adapt-to-shape (ATS) option (median 80, range 53–235 min). Most patients (77%) experienced self-resolving grade 1–2 acute radiation-induced toxicity; none had grade ≥ 3. Three participants (9%) experienced late grade 1–2 toxicity, potentially attributable to SBRT, with one (3%) experiencing grade 3. **Conclusions:** We conclude that MR-Linac-based SBRT, supported by online plan adaptation, is a feasible, safe, and highly precise treatment modality for the definitive management of select upper urinary tract lesions.

## Simple Summary

The MR-Linac (or MRL) is a powerful new device that integrates high-resolution magnetic resonance imaging (MRI) within a linear accelerator to enhance the precision of radiation treatment delivery beyond the predominantly CT-guided standard of care. Our institution was one of the seven founding members of the consortium that tested and refined the 1.5 Tesla MR-Linac in preparation for the first-in-human clinical trials, resulting in several years of early clinical experience. Its application in delivering ablative doses (stereotactic ablative radiation therapy; SBRT) to renal cell carcinoma (RCC) or upper tract urothelial carcinomas (UTUC) has been of particular interest out of clinical necessity and technical challenge. We present a retrospective analysis of our multi-year experience using MRL-SBRT, with emphasis on our evolving treatment setup and early clinical outcomes. Our aim is to contribute to and support the development and innovation of further programs using one of the largest worldwide single-institution cohorts.

## 1. Introduction

Delivering ablative doses of radiation therapy to upper urinary tract targets is a complex endeavor [1,2,3]. It involves the accurate tracking and treatment of a critical and mobile retroperitoneal organ in close proximity to visceral structures [4]. Nonetheless, these significant technical challenges must be addressed, as a substantial proportion of patients cannot undergo gold-standard surgical resection due to medical comorbidities and/or risk of treatment-induced renal insufficiency [5]. 

The integration of magnetic resonance imaging with linear accelerators (MR-Linacs) into the treatment planning and delivery process has shown great promise [6,7,8]. This technology leverages the superior soft tissue contrast and temporal resolution of MRI to enable direct tumor visualization and real-time monitoring of motion throughout the respiratory phases [9]. Such capabilities facilitate daily adaptation and optimization of the treatment plan, ensuring precise targeting of the tumor while sparing surrounding organs at risk (OARs) [10]. Clinical experience with a 0.35-Tesla (T) MR-Linac system has shown encouraging locoregional control and preservation of renal function in the setting of RCC, highlighting the potential of MR-guided SBRT for the effective management of upper urinary tract malignancies (including primary RCC, UTUC, and secondary involvement by other histologies) [11,12]. However, treatment protocols involving daily online plan adaptation and the use of the more advanced 1.5 T MR-Linac system are underdeveloped, and the outcomes from such treatments have yet to be systematically reported. We describe our retrospective, single-institution experience with 1.5 T MR-Linac guided upper urinary tract SBRT, with a focus on key workflow and physics considerations. 

## 2. Materials and Methods

### 2.1. Patient Selection

Eligibility for MR-Linac-guided SBRT was jointly determined by a multidisciplinary genitourinary tumor board, on the basis of expected benefit from consolidative radiotherapy for biopsy-proven disease (or in cases with high clinical and/or radiographic suspicion and perceived high risk for biopsies owing to medical comorbidities, although all patients in this analysis had biopsy-confirmed disease prior to treatment), and target movement of ≤10 mm on a 4D-CT scan while the patient was immobilized with a Vac-Lok cushion and a compression belt (described further below). Additional considerations included whether patients could fit within the MR-Linac body coil, could maintain a consistent position for extended periods, and had no contraindications to MRI (e.g., implanted ferromagnetic materials or pacemakers). This retrospective study was approved by the institutional review board (IRB 2022-0521), and due to the retrospective nature of the research, informed consent was not required. 

### 2.2. Simulation

Treatment simulation was performed with both MRI and CT on the same day to facilitate precise rigid registration. Patients were positioned head-first supine, with arms up and legs on a knee support. For patients who had difficulty holding both arms up, the arm on the contralateral side was positioned down alongside the body. Patients were immobilized with a Vac-Lok cushion. The field of view of the CT scan was set at 600 mm to ensure that the entire body was visible. The default image matrix size was configured to 512 pixels, and the standard slice thickness for all scans was maintained at 3 mm, with a slice spacing of 2.5 mm. A pneumatic compression belt, modified in-house for adaptability, was used to minimize respiratory motion. Its pressure was adjusted for each patient for comfort, without compromising the effectiveness of the immobilization during simulation.

MR images were obtained with a Unity MR-Linac system (Elekta AB, Stockholm, Sweden) [13], which is based on a Philips 1.5 T Marlin MRI and includes a four-element anterior coil and a built-in four-element posterior coil to provide coverage of the abdominal region. Motion evaluations were performed with cine-mode MRI imaging (a 2D balanced turbo fast echo [TFE] dynamic MR scan; details in Table 1) during the MR simulation to quantify cranial–caudal tumor motion both, with and without use of the compression belt. Initially, MR images were captured without a compression belt, and then patients were fitted with a compression belt to apply a tolerable level of pressure. The cine MR image was aligned to the center of the target in both scenarios, and cine image acquisition lasted approximately 1 min. The motion range of the target was measured by using Philips image tools from the console, along with the cine images. After that, a T2-weighted 3D sequence and a T1-weighted 3D VANE mDixon sequence with fat suppression were acquired for the treatment planning system (Table 1). The 4D CT scans were then obtained with the belt in place to verify the motion consistency and to establish the final motion range.

To assess motion reduction and its consistency from simulation to daily treatment sessions, daily cine MR images, with the same MR sequence used in simulation, were captured and analyzed for each treatment fraction. 

### 2.3. Treatment Planning 

The Monaco treatment planning system (Elekta, Inc. Maryland Heights, MO, USA) was used to create reference radiotherapy plans based on the primary CT scan, according to planning directives established by the Genitourinary Radiation Oncology Service at MD Anderson Cancer Center. The GTV was drawn from the maximum intensity projection (MIP) of the 4D-CT scans as volume encompassing the entire tumor, with a subsequent 5 mm isometric expansion of the GTV used to derive the planning target volume (PTV). The clinical goals were to provide at least 100% coverage of the GTV and 95% coverage of the PTV by using prescribed doses of 36 Gy (7 patients; 21%), 39 Gy (1 patient; 3%), or 42 Gy (26 patients; 76%), given in three fractions, every other day. The OAR constraints are summarized in Table 2. Before treatment was delivered, we performed a secondary monitor unit (MU) check by using RadCalc (Version 6.3, Lifeline Software Inc., Austin, TX, USA) and an intensity-modulated radiation therapy (IMRT) QA measurement using ArcCheck MR (Sun Nuclear Corporation, Melbourne, FL, USA) to ensure the accuracy and deliverability of the generated plan. 

### 2.4. Online Plan Adaptation and Treatment Delivery 

Patients were positioned on the MR-Linac couch using a pre-established index value from the simulation that guided their longitudinal placement. Because the MR-Linac lacks external lasers, therapists used an internal sagittal laser and leveling markers on each patient’s skin to ascertain lateral positioning and to mitigate any potential body rotations. After setup, a T1 3D mDixon (water phase) MRI scan was obtained and subsequently fused with the reference plan image for plan adaptation. Plans could be adapted by using one of two strategies provided by the MR-Linac system: “adapt-to-position” (ATP) or “adapt-to-shape” (ATS) [13,14]. 

The ATP workflow involves rigid registration of the daily MRI to the reference CT to calculate positional shift and update the treatment isocenter of the plan. In ATP, no contour modifications are performed; instead, the daily adaptive plan is either recalculated or reoptimized directly from the reference plan to account for the isocenter shift and to maintain dose consistency. This workflow is most effective when anatomical changes between fractions are minimal, ensuring that the precision and conformity of dose delivery are maintained. ATP is similar to traditional image-guided radiotherapy (IGRT), utilizing daily MRI for alignment and treatment guidance, relying on reference CT anatomy for dose calculations. The ATS workflow uses deformable image registration (DIR) to propagate contours from the reference CT to the daily MRI, followed by manual adjustments if needed to reflect daily anatomical changes. A comprehensive re-optimization of the treatment plan is conducted based on the updated anatomy captured by the daily MRI, allowing for greater flexibility in dose distribution. ATS is particularly suitable for situations involving substantial anatomical changes, such as tumor shrinkage, weight loss, or shifts in the position of organs at risk. This approach is analogous to creating a new adaptive CT simulation and treatment plan, offering a higher degree of adaptation to meet the patient’s current anatomical configuration.

The ATP workflow was chosen for every patient by default. The ATS workflow was chosen based on the following criteria: (1) substantial anatomic changes in nearby GI organs requiring recontouring to evaluate dose; (2) the OAR dosimetric goals (e.g., bowel dose) could not be met; and (3) changes in tumor size to an extent requiring CTV recontouring.

Before radiation delivery, each adaptive plan underwent an MU verification by using the RadCalc system. During beam delivery, internal anatomic motion was monitored in real time by using three orthogonal cine MR images centered at the tumor. At the completion of each fraction, an additional IMRT QA assessment was conducted on the adapted plan for the ATS workflow, serving as an extra measure of quality control.

## 3. Results

### 3.1. Adaptive Plan and Treatment Delivery 

From August 2020 through April 2024, 34 patients were successfully treated with 1.5 T MR-Linac-based SBRT at MD Anderson Cancer Center. Only one patient (3%) did not complete the final fraction because of unresolvable logistic considerations, receiving 28 Gy of the prescribed 42 Gy; the other 33 patients successfully completed the intended treatment. 

**Figure 1:**
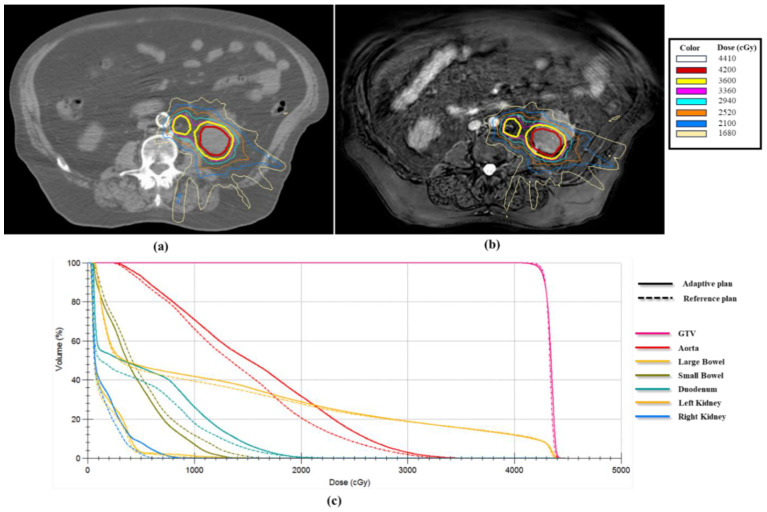
Illustration of an online adaptive plan for MR-guided SBRT. (**a**) Reference plan shown on the simulation CT image. (**b**) The adaptive plan for the 3rd fraction shown on the daily MR image. (**c**) The dose-volume histogram (DVH) comparing the reference plan (dashed lines) and the adaptive plan (solid lines).

The [default] ATP workflow was used for most of the treatment fractions for all 34 patients. Nevertheless, a combination of online ATP and online ATS was required for 10 patients, with a minimum of one ATS during the treatment course to account for GI dose constraints. The adaptive plans were critical to meet the dosimetric goals set in the reference plans, ensuring precise dose delivery in these 10 patients. Figure 1 shows one example of an ATS plan dose overlaid on the daily MR image and compared with the reference plan dose. It was observed that the interfraction anatomical variability, particularly within the duodenum, required ATS in this specific case to maintain target dose and OAR sparing. 

Across the treatment cohort, 102 fractions were planned, with 101 successfully delivered via MR-Linac. The single undelivered fraction resulted from a patient’s decision to decline the final treatment session. Of the delivered fractions, 88 utilized ATP and 13 employed ATS, reflecting the specific needs for adaptation. The median duration of treatment was 56 min overall and was significantly shorter with ATP (median 54 min, range 38–97 min) than ATS (median 80, range 53–235 min). 

### 3.2. Motion Management with the Compression Belt 

**Figure 2:**
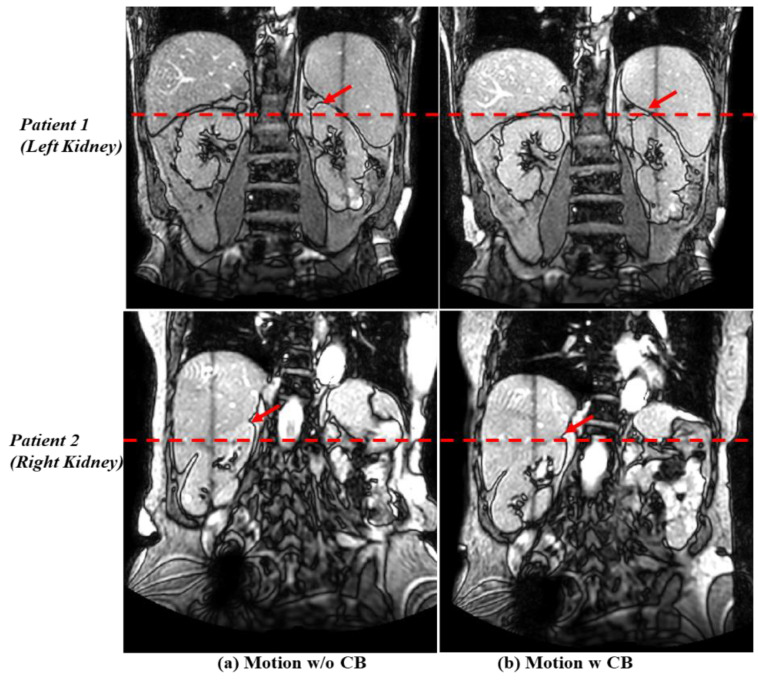
Examples of kidney target motion management with a compression belt (CB) in two patients undergoing magnetic resonance-guided stereotactic body radiation therapy. (**a**) Tumor motion without use of the belt was 18 mm for Patient 1 (top) and 13 mm for Patient 2 (bottom). (**b**) Tumor motion was significantly reduced with use of the belt to 7 mm for Patient 1 (with a left kidney tumor) and to 3 mm for Patient 2 (with a right kidney tumor), as shown by the dashed red lines and arrows.

The pneumatic compression belt was introduced into routine clinical practice for motion management of abdominal cancers to be treated with MR-Linac starting in July 2023. Before that, only 4D-CT was acquired for the tumor motion evaluation, without the compression belt. Tumor motion control with the compression belt in place was analyzed for 19 patients (9 with left and 10 with right kidney tumors); two examples are shown in Figure 2.

**Figure 3:**
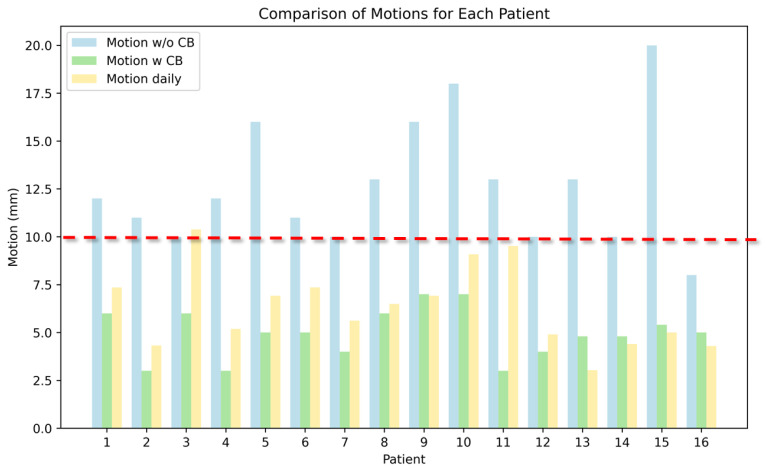
Comparison of respiratory-induced target motion in 16 patients, with and without use of a compression belt (CB) during MR-guided SBRT. Blue indicates cranio–caudal motion without the belt; green shows motion with the belt; and yellow indicates the average daily motion with the belt applied during treatment sessions. The red dashed line represents the clinical threshold of 10 mm, below which motion is considered sufficiently controlled to allow MR-Linac treatment. Although some patients experienced greater daily motion, it generally fell below the clinical thresholds for treatment, particularly if a belt was used during simulation.

Of these 19 patients, 16 (75%) displayed substantially reduced tumor motion with the compression belt. Mean cranial–caudal motion decreased from 12.1 mm (range 8–20 mm) without the belt to 5.0 mm (range 3–7 mm) with the belt (a mean 59% motion reduction). Indeed, the reduction in motion to <10 mm allowed these patients to undergo MR-Linac treatment. Daily motion assessment demonstrated a consistent mean motion of 6.3 mm (range 2.5–10.4 mm) when the belt was used. Of the 19 patients, one could not tolerate the belt, and tumor motion for two others could not be reduced to <10 mm, and those 3 patients were not treated with MR-Linac. Tumor motion for the 16 patients treated successfully with the compression belt and the MR-Linac is shown in Figure 3.

### 3.3. Patient Outcomes 

A total of 25 (73%) participants were treated for an upper urinary tract primary malignancy, 6 (18%) for an upper tract malignancy in a solitary kidney, and 3 (9%) for a non-genitourinary malignancy in the upper urinary tract. A total of 26 patients (77%) experienced an acute toxicity, and 4 (12%; one patient was lost to long-term follow-up) developed a late, likely radiation-attributable, toxicity per the Common Terminology Criteria for Adverse Events (CTCAE) v5.0. Acute toxicities included one or a combination of grade 1–2 fatigue (46%), nausea (31%), pain (abdominal, tumor, or chest wall; 15%), vomiting (4%), and/or diarrhea (4%), and these were typically self-resolving. Among long-term toxicities, patients described grade 1–2 nausea (18%), constipation (18%), fatigue (9%), diarrhea (9%), pain (abdominal; 9%), and irritative urinary symptoms (frequency; 9%); there was one instance (9%) of grade 2 hematuria with associated grade 2 urinary tract obstruction (9%); one patient (9%) developed possible treatment-related grade 3 pyelonephritis (reported as kidney infection as the most similar CTCAE v5.0 category). This was treated with brief hospitalization for observation and administration of intravenous antibiotics. No patient experienced treatment-related death or kidney failure, despite the aggressive application of SBRT.

## 4. Discussion

We report our single-institution clinical experience with using a 1.5 T MR-Linac to plan and deliver SBRT for either the primary management of RCC or UTUC or the consolidation of secondary kidney involvement by metastatic disease. To our knowledge, this is the largest series describing the technique, feasibility, and safety of this approach. 

The seminal Multi-OutcoMe EvaluatioN of radiation Therapy Using the MR-Linac (MOMENTUM) study opened in 2019 as a multi-national collaborative database collecting clinical and technical data of patients treated on the Unity MR-Linac [15]. To date, only 24 participants have been included due to the challenges in patient recruitment, with most centers contributing <5 and just one having treated >10 individuals. Nonetheless, this initiative is imperative in providing an infrastructure for multicenter outcome reporting and highlights the importance of this study in contributing to the limited evidence base.

MR-guided radiotherapy provides superior soft-tissue contrast with enhanced visualization of the target and adjacent OARs, a capability particularly useful in the management of lesions within the upper urinary tract, which are moving and are in proximity to several radiosensitive gastrointestinal structures. Our 1.5 Tesla MR-Linac system enabled a high degree of confidence in achieving a conformal dose distribution while simultaneously meeting surrounding OAR dose constraints. It holds the potential to substantially contribute to the efficacy of SBRT and may enable further dose escalation, while mitigating the risk of toxicity. In our experience, the implementation of a pneumatic compression belt for patients receiving MRL-SBRT had a pivotal role in managing intrafraction motion due to respiration. It significantly reduced the cranial–caudal movement of the tumor (from 12.1 mm to 5.0 mm), thereby allowing for more stable, reproducible, and precise treatment delivery, critical components in SBRT. However, while most patients tolerated the compression belt well, a small subset required alternative motion management strategies, underscoring the need to develop additional approaches. All of the included patients were treated before comprehensive motion management (CMM) [16] was available in our Unity system. With CMM respiratory gating, the compression belt may still be useful to reduce motion and improve the duty cycle of gating treatment. The use of the compression belt is not limited to MR-Linac treatment. We have established the protocol of using the compression belt for general motion management of thoracic and abdominal cancer treatments subject to respiratory motion when breath-hold or other motion management approaches are not an option [17,18,19]. The compression belt is particularly useful for MR-Linac treatment before the CMM is available in the MR-Linac. The use of the compression belt helped enhance the precision of the dose delivery to moving targets, contributing to improved clinical outcomes by reducing radiation exposure to healthy surrounding tissues.

A notable concern with the use of the MR-Linac is the total treatment duration. Recorded median treatment time was 56 min. Fractions requiring ATS significantly extended the median treatment time to 80 min, compared with 54 min for those using solely ATP. This increased duration for ATS was due to the extensive processes required to propagate contours from the reference CT to the daily MR images, followed by thorough verification by the radiation oncologist. However, implementation of adaptive planning is critical in this application, as upper urinary targets are highly susceptible to daily anatomical changes and are located near critical structures like the bowel [20,21]. Although adaptive planning results in longer treatment times, the benefits of increased precision and safety are substantial. The anticipated integration of auto-contouring [22] and optimized workflows [9,23], as well as ongoing developments in automation and AI-driven processes, are expected to significantly reduce the time and resource demands of ATS strategies, thereby enhancing the usability of MRL-SBRT [24]. These advancements will reduce the need for specialized training and mitigate the challenges associated with longer treatment durations, making MR-Linac technology a feasible and efficient option for a broader range of clinical settings, including small centers with limited personnel, resources, and expertise [25].

Most patients (77%) experienced self-resolving grade 1–2 acute toxicities, with just four experiencing late toxicities of grades 1–2 (3 patients; 9%) and grade 3 (1 patient; 3%). These results are encouraging considering the 10% rate of grade 3 treatment-related adverse events (nausea and vomitting; abdominal, flank or tumour pain; colonic obstruction; and diarrhea) within TROG15.03 FASTRACK II and the aggregate grade 2 (5.3%) and grade 3 (2.7%) treatment-related effects reported in the systematic review and practice guidelines from the ISRS. We did not experience any grade 4+ toxicities as were reported in 1% of patients (gastritis, duodenal ulcer) in the 5-year update of the individual level meta-analysis from the IROCK group, nor did any patients require dialysis (reported previously as occuring in 1–4% of patients), despite the aggressive application of SBRT [26,27,28]. 

This analysis exhibits several strengths, as well as some limitations. It provides the largest single-institution experience with 1.5 T MRI-Linac-based SBRT for the treatment of upper urinary tract targets. Prior publications have reported outcomes from 0.35 T MRL SBRT for primary RCC [12] or have investigated the application of 1.5 T MRL in the management of upper abdominal [29] and prostate primary malignancies [30]. Moreover, nearly all patients were contoured and treated by two radiation oncologists at MD Anderson, using doses of 36–42 Gy in three fractions, and their final treatment plans underwent comprehensive intradepartmental peer review prior to delivery. Among the limitations of this study to be considered include first, the single-institution, retrospective nature of the analysis, which included patients from across the United States, and physician-recorded toxicities, carrying the potential for underreporting or not reporting toxicities experienced. 

## 5. Conclusions

This study contributes to the workflow and evidence base supporting the safe use of 1.5 T MR Linac SBRT as a definitive treatment strategy for upper urinary tract malignancies occurring in patients who are precluded from surgical resection. The superior soft tissue discrimination, treatment monitoring potential, and precision of the MR-Linac-based approaches make them well suited to treating these complex lesions. Despite the promising outcomes, this single-institution, retrospective analysis is limited by the potential underreporting of toxicities. Future multicenter studies with extended follow-up are needed to further validate these results. Nevertheless, MR-Linac technology represents a significant advancement in precision radiotherapy, offering an effective, definitive treatment modality with the potential for dose escalation and enhanced tumor control, while minimizing toxicity.

## Figures and Tables

**Table 1:** Parameters and acquisition techniques for MR imaging during MR simulation and treatment sessions.

Sequence	T2w 3D	T1w 3D VANE mDixon	Cine MRI for Simulation	Cine MRI for Daily Treatment
Scan Technique	Spin echo	Gradient echo	Gradient echo	Gradient echo
Imaging Mode	Turbo SE	Radial FFE	Balanced TFE	Balanced TFE
Imaging Orientation	Axial	Axial	Sagittal, Coronal	Axial, Sagittal, Coronal
Acquisition Type	3D	3D	2D	2D
Image Contrast	T2	T1	T2/T1	T2/T1
Repetition Time (ms)	1300	6.3	4.3	3.8
Echo Time (ms)	87	1.9/3.6	2.2	1.9
Flip Angle (°)	90	10	40	40
Pixel Bandwidth (Hz)	693	857	478	1085
Echo Train Length (ETL)	90	2	68	48
Radial Oversampling	NA	225	NA	NA
Field of View (cm^3^)	48 × 48 × 25	45 × 45 × 24	51 × 51 × 0.6	44 × 44 × 0.5
Voxel Size (mm^3^)	0.83 × 0.83 × 1	0.78 × 0.78 × 1.5	0.96 × 0.96 × 6	1.3 × 1.3 × 5
Sense Factor	4	1.3	3	3
Fat Saturation	None	Dixon	None	None
Number of Averages	2	1	1	1
Dynamic Times	1	1	100	>1500
Scan Time (minutes)	4:00	4:30	1:04	>15:00

**Table 2:** Dose constraints for organs at risk for kidney stereotactic body radiotherapy in three fractions.

	36–42 Gy Plans
Liver	D700 cm^3^ ≤ 15 Gy
	Dmean ≤ 16 Gy
Spinal Cord	Dmax ≤ 18 Gy
	D10 cm^3^ ≤ 15 Gy
Contralateral Kidney	V10 Gy ≤ 10%
Small Bowel	Dmax ≤ 28 Gy
	V12.5 Gy ≤ 30 cm^3^
Large Bowel	Dmax ≤ 38 Gy
	V35 Gy ≤ 1 cm^3^
	V30 Gy ≤ 10 cm^3^
Duodenum	Dmax ≤ 28 Gy
Stomach	V22.5 Gy ≤ 4 cm^3^
	Dmax ≤ 28 Gy
Target Kidney-GTV	V10 Gy ≤ 50% (Optional)
Chest Wall	D30 cm^3^ ≤ 30 Gy (Optional)

## Data Availability

The dataset is available through the MOMENTUM clinical trial.
